# A Bidirectional Neuromodulation Technology for Nerve Recording and Stimulation

**DOI:** 10.3390/mi9110538

**Published:** 2018-10-23

**Authors:** Jian Xu, Hongsun Guo, Anh Tuan Nguyen, Hubert Lim, Zhi Yang

**Affiliations:** 1 Department of Biomedical Engineering, University of Minnesota, 312 Church Street SE, Minneapolis, MN 55455, USA; xuxx1268@umn.edu (J.X.); guoxx691@umn.edu (H.G.); nguy2833@umn.edu (A.T.N.); hlim@umn.edu (H.L.); 2 Department of Otolaryngology, Head and Neck Surgery, University of Minnesota, 516 Delaware Street SE, Minneapolis, MN 55455, USA; 3 Institute for Translational Neuroscience, University of Minnesota, 2101 6th Street SE, Minneapolis, MN 55455, USA

**Keywords:** system-on-chip, neuromodulation, bidirectional, closed-loop, sciatic nerve, vagus nerve, precision medicine

## Abstract

Electrical nerve recording and stimulation technologies are critically needed to monitor and modulate nerve activity to treat a variety of neurological diseases. However, current neuromodulation technologies presented in the literature or commercially available products cannot support simultaneous recording and stimulation on the same nerve. To solve this problem, a new bidirectional neuromodulation system-on-chip (SoC) is proposed in this paper, which includes a frequency-shaping neural recorder and a fully integrated neural stimulator with charge balancing capability. In addition, auxiliary circuits consisting of power management and data transmission circuits are designed to provide the necessary power supply for the SoC and the bidirectional data communication between the SoC and an external computer via a universal serial bus (USB) interface, respectively. To achieve sufficient low input noise for sensing nerve activity at a sub-10 μV range, several noise reduction techniques are developed in the neural recorder. The designed SoC was fabricated in a 0.18 μm high-voltage Bipolar CMOS DMOS (BCD) process technology that was described in a previous publication and it has been recently tested in animal experiments that demonstrate the proposed SoC is capable of achieving reliable and simultaneous electrical stimulation and recording on the same nerve.

## 1. Introduction

Electroceuticals is a new research area in bioelectronics that aims to create implants, including chips that are as small as a grain of rice, and the implants are expected to simultaneously monitor and modulate nerve activity to treat diseases and augment or replace drugs [1,2,3,4]. With these implants, electrical stimulation is combined with low-noise and artifact-free recording, enabling closed-loop neuromodulation therapies for a variety of chronic disorders including hypertension, heart failure, gastrointestinal disorders, type II diabetes, and inflammatory disorders [5,6]. The effectiveness of electroceutical therapy can be monitored and optimized for each patient by applying controlled amounts of charge into the nerve while monitoring the neural responses from the treated organ or system at the same time. For example, vagus nerve stimulation is emerging as an alternative method for treating multiple health disorders, such as seizures, depression, rheumatoid arthritis and tinnitus. To minimize side effects and optimize treatment, the stimulation levels could be titrated to the lowest levels required to elicit the desired spiking activity along the ascending and/or descending vagus nerve and also to elicit specific firing patterns in the nerve relevant for treatment. Another example is in providing better neural prosthetics to amputees for controlling robotic hands by monitoring motor and sensory neural activity from ulnar and median nerves to control the hand movements as well as electrically stimulate the nerves to restore sensory sensations and feedback based on the recorded signals.

Highly effective electroceuticals are not in currently use because state-of-the-art bioelectronics do not have three critical features [7,8,9,10]. First, existing neural recording technologies have sufficient noise characteristics for sensing action potentials in brain recordings, but this is inadequate for resolving small neural signals from noise sources in cuff electrode interfaces on nerves [11]. Second, penetrating electrodes can improve the signal quality by recording closer to the nerve fiber, but the signals typically decay over time due to the foreign body response caused by electrode penetration and damage of the nerve as well as the micro-motion of the tethered electrodes relative to the soft nerve tissue [12]. Third, electrical stimulation produced by adjacent electrodes generates large noise and recording artifacts on the same nerve that can be multiple orders of magnitude larger than the spontaneous or evoked nerve activity, making it extremely challenging to perform simultaneous recording and stimulation on the same nerve [13].

In this paper, a neuromodulation system-on-chip (SoC) is proposed for nerve recording and stimulation. We have recently pioneered a new neural recorder architecture that does not need built-in analog filters, thus avoiding the filter noise that is a major noise source in nerve recordings [14,15,16,17,18]. We also propose new techniques that can significantly reduce activity-dependent noise (shot noise) in the electrode interface, which is another primary noise source. The combination of “filterless” amplification and shot noise suppression permits an entirely different approach to improve signal quality at a sub-10 μV range. Thus, we aim to develop an ultra-low-noise recording chip for sensing nerve activity at a sub-10 μV range, even with electrical stimulation on the same nerve. We will test and use the SoC in animal experiments to monitor nerve activity in the presence of stimulation artifacts.

The paper is organized as follows. The research background and limitations in the nerve recording technology field are discussed in Section 2. Section 3 presents the design of the proposed bidirectional neuromodulation system that was described in detail in the previous publications [16,18,19] and reviewed in this paper. New animal experimental results obtained with a prototype of our neuromodulation system are presented in Section 4. Section 5 summarizes this paper.

## 2. Background and Limitations

It is well known that the ability to simultaneously stimulate and record nerve activity is currently limited and that new technologies are required to provide high-fidelity chronic recording and stimulation in animals for basic science studies and, eventually, for human clinical applications. In particular, there are three limitations that prevent chronically stable nerve recording and stimulation.

Limitation 1: There is poor signal-to-noise ratio (*SNR*) of recorded signals on nerves. Epineural electrodes such as cuff electrodes provide a robust and stable interface for recording whole nerve activity, but the electrical isolation caused by the epineurium/perineurium reduces the magnitude of detected signals, which translates into low *SNR* [20,21]. The main reasons for low *SNR* are given as follows. (1) The biological noise in epineural electrodes can significantly distort recordings and require heavy filtering. Due to insufficient recording precision and the difficulty of resolving nonstationary signals and noise through filtering, residual biological noise can be intermixed with the desired nerve signals. (2) Part of the electrode-tissue interface noise is ohmic that in principle cannot be separated from signals. However, several models and more recent measurement data [22,23,24] suggest that a large portion of electrode noise is non-ohmic. For example, [25] compared an electrode with 100-fold increased surface area that should have reduced the ohmic noise by ten-fold, but the measured total electrode noise was only reduced by 40%. (3) The amount of electronic noise in nerve recording can be quite large due to elevated filter noise. When recording from a nerve in the abdomen, for example, the constant motion of viscera guarantees large amplitude motion artifacts. To sufficiently attenuate motion artifacts to avoid saturating electronics, a built-in analog high-pass filter with a corner frequency of at least ten times higher than the artifact frequency is required. This requirement increases the analog filter noise over the neural signal band to tens of μV and beyond. In some other cases, circuits and devices that have low-noise characteristics in benchtop testing do not work well in actual experiments. For example, [26,27,28] have reported a neural interface based on epineural electrodes and low-noise neural amplifiers [11,29], where the measured *SNR* on a sciatic nerve preparation is only 1–3 dB, which is not high enough to isolate the small nerve signals.

Limitation 2: There is an inability to record microvolt-level nerve activity in the presence of stimulation pulses applied to the same nerve. The stimulation artifacts often produce a shift in the differential mode signal and the common mode signal, both of which need to be properly rejected. The subtraction of stimulation artifacts requires compensating for the frequency-dependent amplitude and phase of the transfer function [8]. This subtraction cannot be routinely done because it requires a high precision amplifier buffer to store superimposed artifacts and neural signals, and a fast recovery from stimulation artifacts. We did a survey on recent recording electronics and found that every extra bit of increased precision requires a four-fold increase in the supply power [30,31,32,33,34,35]. In other words, an 8-bit increase in precision translates into a >60,000 times increase in power. Thus, it is not possible to implant a 10 W nerve recorder into the body. Furthermore, fast recovery from stimulation artifacts is another challenge. Electrical stimulation saturates the recorder and increases the noise floor in addition to creating “ringing” in the signals that only slowly stabilizes towards zero. The closer the stimulation and recording electrodes, the more artifacts and noise appear in the recorded signals. As a result, a typical nerve experimental setup requires direct electrical stimulation of a portion of the nerve and recording of the nerve at another distant location. Therefore, current technologies described in the literature cannot yet support simultaneous recording and stimulation on the same nerve.

Limitation 3: There is an inability to extract signals of individual fascicles with current noninvasive electrodes. Considerable research effort has been devoted to developing and improving neural interfaces using a variety of designs and materials. Noninvasive nerve interface approaches provide small signals from a highly limited number of electrodes. Current technologies have demonstrated successful recording from the sciatic nerve, where the recorded compound action potentials (CAPs) are sufficiently greater than the background noise activity [36,37]. However, when using a noninvasive wire or cuff electrode, it is still not readily possible to acquire more resolved neural activity from the sciatic nerve or other peripheral nerves (i.e., representing nerve fibers or small groups of fibers that is possible with penetrating electrodes) using current commercial devices.

In this paper, a bidirectional neuromodulation SoC is proposed for simultaneous nerve recording and stimulation. With several new techniques that will be presented in Section 3, the proposed SoC is supposed to be able to overcome many of the limitations described above.

## 3. Bidirectional Closed-Loop Neuromodulation System Design

**Figure 1:**
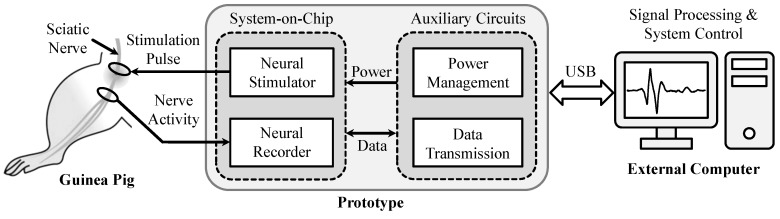
An illustration of the proposed bidirectional neuromodulation system for nerve recording and stimulation experiments.

Figure 1 shows an exemplary bidirectional neuromodulation system for supporting simultaneous recording and stimulation without one impeding the other. The neuromodulation system includes a SoC and auxiliary circuits, where the SoC consists of a fully integrated neural recorder and a electrical neural stimulator that are implemented in a high-voltage Bipolar CMOS DMOS (BCD) process technology, and the auxiliary circuits that comprise power management and data transmission circuits are implemented using consumable components. The operation of the SoC is facilitated by the customized auxiliary circuits. In this design, the function of the power management circuits is to provide the required power supply and voltage references for the SoC. The function of the data transmission circuits is to provide the bidirectional data communication between the SoC and an external computer via a customized universal serial bus (USB) interface, which is developed for adjusting system parameters, such as total loop gain, data acquisition bandwidth, stimulation waveforms & patterns, stimulation rate, stimulation current/voltage, etc. In order to perform simultaneous nerve recording and stimulation experiments, both the neural recorder and neural stimulator are connected to the same sciatic nerve of a guinea pig.

### 3.1. Neural Recorder Design

**Figure 2:**
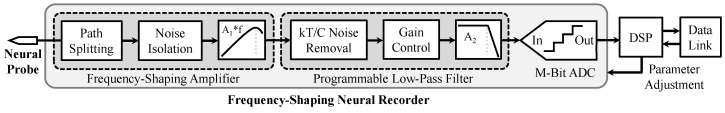
Block diagrams of the proposed neural recorder.

For the neural recorder design, we have previously presented a frequency-shaping (FS) technique [17] that can remove electrode offset without requiring a sub-Hz high-pass filter, increase input impedance by 5–10-fold, compress neural data dynamic range, and support full bandwidth recording up to several kHz [14,16]. Figure 2 gives the detailed block diagrams of the proposed recorder that is developed for nerve recording experiments, which includes a frequency-shaping amplifier (FSA) stage with noise optimization techniques, a programmable low-pass filter stage, an M-bit analog-to-digital converter (ADC), and a digital signal processor (DSP). The FSA stage has a frequency dependent gain characteristic such that artifacts appearing at low frequencies are attenuated and nerve activity at high frequencies is amplified, and the low-pass filter is used to remove high-frequency noise outside the signal bandwidth. After the recorded signals are digitized by the ADC, a matched digital filter is then applied to reconstruct data supposedly observed at the electrode and the reconstruction data are passed to the DSP for information decoding.

To substantially reduce input noise floor at low frequencies, one noise reduction technique of path splitting is developed in the FSA stage to generate two separate amplification pathways for low-frequency (1–300 Hz) and high-frequency (300–5000 Hz) signal acquisition. Also, a noise isolation method is added in the FSA stage to avoid charge transfer from parasitic capacitor $C_{p}$ to the feedback capacitor $C_{f}$ for further noise optimization. To reduce parasitic capacitors, all the switches and amplifier input-pair transistors are designed with small size, and the amplifier input-pair transistors are biased in the sub-threshold region. In addition, it is well known that switched-capacitor circuits usually bring in kT/C noise caused from switch-on resistance. In the proposed neural recorder, to achieve high input impedance, the value of $C_{f}$ is set to be tens of fF, thus large kT/C noise will appear on $C_{f}$ after the reset switch is turned off. Fortunately, the kT/C noise on $C_{f}$ is generated during resetting phase and remains constant during amplification phase. Given the noise generation is not directly correlated with the signal transfer, an auto-zero kT/C noise cancellation scheme is proposed to reduce the noise on $C_{f}$ with three steps: reset the charge, sample the kT/C noise, and remove the kT/C noise.

To extend the signal application range of the designed recorder, the recording bandwidth, bias current, and sampling frequency are designed to be adjustable from 1–625 Hz to 1–5000 Hz, 25 nA to 200 nA, and 5 kHz to 40 kHz, respectively. More details about circuit implementation of the proposed recorder are presented in [16,18].

### 3.2. Neural Stimulator Design

In our device design, we propose an integrated, current-mode microstimulator that can support high voltage compliance and high output impedance, where the output waveform, current, timing, and pattern are fully programmable for each channel in real time [19]. Both passive and active charge-balancing schemes are implemented to reduce the residual voltage and its accumulation over time, which are important safety features, especially for chronic applications.

**Figure 3:**
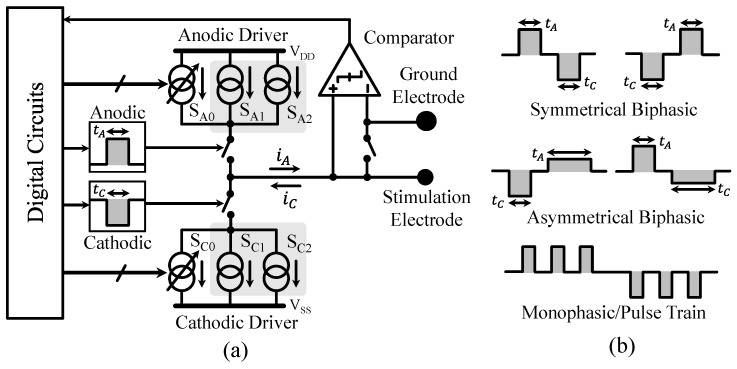
(**a**) Simplified functional block diagrams of one stimulator channel; (**b**) Examples of stimulation waveforms and patterns used in the experiments. Reproduced with permission from Anh Tuan Nguyen, A Programmable Fully Integrated Microstimulator for Neural Implants and Instrumentation; published by IEEE Biomedical Circuits and Systems Conference (BioCAS), 2016.

Figure 3a shows the simplified diagrams of one stimulator channel that consists of three major functional blocks: current drivers, digital circuits, and charge-balancing circuits. Each current driver includes two matched sub-drivers, namely ($S_{A1}$, $S_{A2}$) and ($S_{C1}$, $S_{C2}$), which can be independently controlled to deliver flexible stimulation waveforms. Two charge-balancing schemes are integrated into the stimulator to remove residual charge on the electrode. In the passive scheme, the output is connected to the ground electrode through a switch. In the active scheme, a comparator is used to monitor the electrode voltage after each stimulus and digital circuits are then employed to adjust stimulation parameters accordingly, including relative anodic/cathodic timing ($t_{A}$, $t_{C}$) and current ($S_{A0}$, $S_{C0}$). Figure 3b also shows examples of stimulation waveforms and patterns used in the experiments, which demonstrate the capabilities of the proposed stimulator for producing a broad range of current waveforms. The stimulator is programmed through a single-wire customized communication protocol. Additionlly, the controller utilizes a 32-bit data frame at 1 Mbit/s, where the first 16-bit encodes the channel identification and the second 16-bit is the instruction set. Thus, the controller can support $2^{16}$ independent channels and $2^{16}$ distinct commands. Stimulation parameters are loaded when the device is powered on. The stimulation waveforms and patterns are then automatically generated by the internal clock generator. During normal operation, individual parameters can be reprogrammed in real time with a 32 μs latency by sending the appropriate commands to the device.

### 3.3. Auxiliary Circuits Design

**Figure 4:**
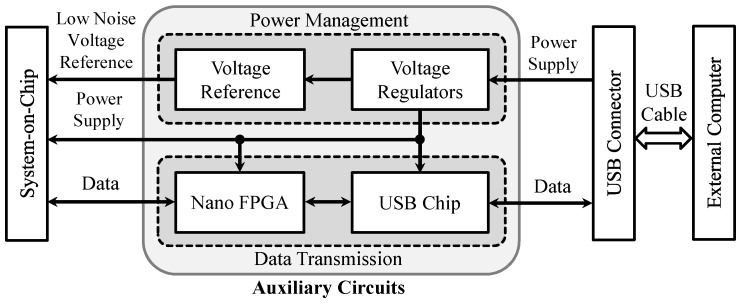
Block diagrams of the auxiliary circuits, which include a nano flash field-programmable gate array (FPGA), a universal serial bus (USB) interface chip, a voltage reference chip, and several voltage regulators.

Figure 4 presents the detailed block diagrams of the auxiliary circuits that include a nano low power flash field-programmable gate array (FPGA) (AGLN250-CS81, Microsemi Corporation, Aliso Viejo, CA, USA), a USB chip (FT245R, Future Technology Devices International Ltd., Glasgow, UK), ultra-low noise voltage reference circuits (ADR445, Analog Devices, Norwood, MA, USA; ADA4896-2, Analog Devices), and several low noise voltage regulators (ADP222, Analog Devices; LTC3260, Linear Technologies, Milpitas, CA, USA). The digital output data of the designed SoC are sent to the nano FPGA for signal processing and information encoding. Afterwards, the FPGA output data are transferred to the external computer using a USB to parallel first-in first-out (FIFO) interface. Meanwhile, control signals from the external computer can be sent back to the SoC for adjusting the system parameters through the same data transmission protocol. Thus, the designed data interface can provide bidirectional communication between the SoC and the external computer. Note that the prototype as shown in Figure 1 is also powered by the external computer through the USB cable. In the power management circuits, the voltage regulators are used to generate different power supplies (1.8 V, 3.3 V, and ±5 V) for both the SoC and auxiliary circuits, and the voltage reference circuits are designed to provide ultra-low noise voltage references (0 V, 0.9 V, and 1.8 V) for the SoC.

## 4. Experimental Prototype and Animal Experimental Results

### 4.1. Experimental Prototype

**Figure 5:**
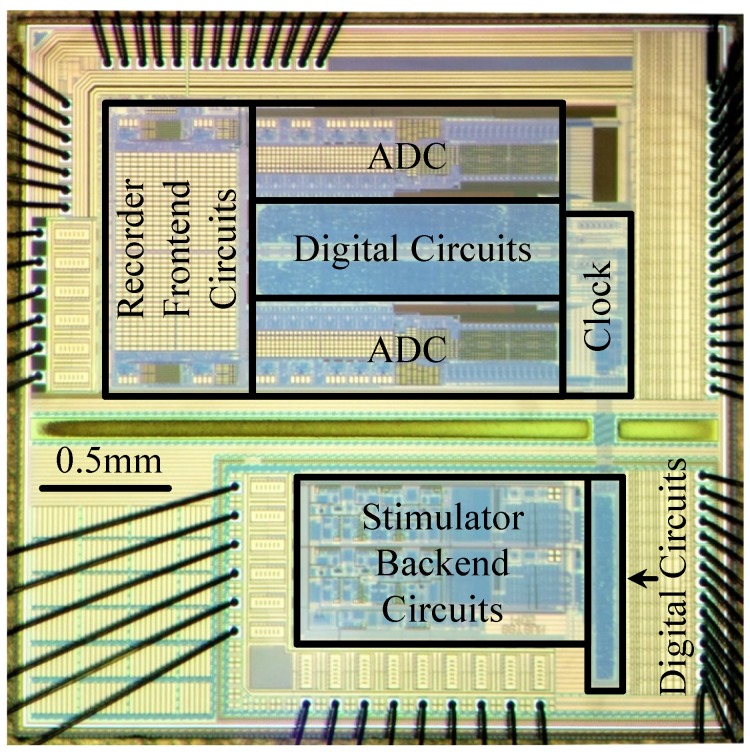
Chip microphoto of the designed neural recorder and neural stimulator in a high-voltage 0.18 μm Bipolar CMOS DMOS (BCD) process.

A prototype SoC including both neural recorder and stimulator was fabricated in a one-poly six-metal (1P6M) high-voltage (HV) 0.18 μm BCD process, and the chip micrograph is given in Figure 5. The core area of the designed two-channel neural recorder is 2.2 mm × 1.2 mm, where the analog frontend circuits consisting of an FSA stage and a programmable low-pass filter stage occupy a circuit area of 500 μm × 600 μm per channel, ADC occupies a circuit area of 1300 μm × 350 μm per channel, digital circuits occupy a circuit area of 1300 μm × 500 μm, and clock generator occupies a circuit area of 250 μm × 720 μm. The core area of the designed two-channel neural stimulator is 1.4 mm × 0.65 mm, where the backend current drivers utilize high-voltage (5 V and 20 V) transistors, digital circuits adopt low-voltage (1.8 V) transistors, and level shifters are added between the stimulator current drivers and digital circuits.

**Figure 6:**
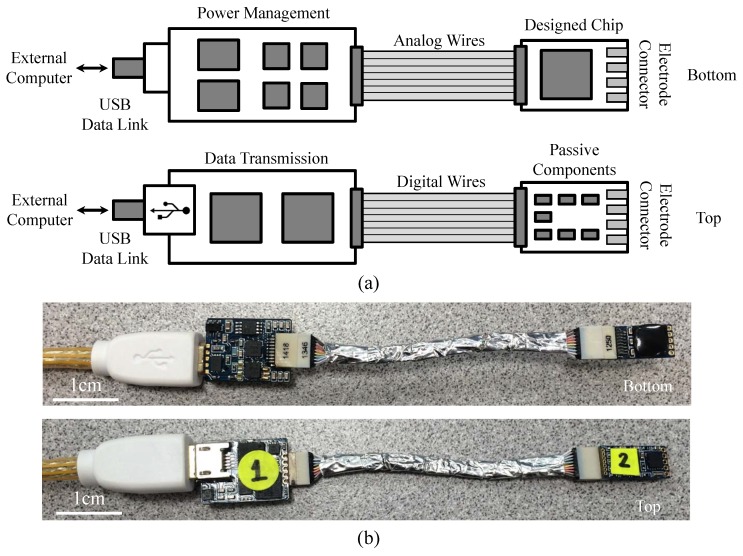
(**a**) Layout illustration (bottom and top side) of the designed neuromodulation system prototype; (**b**) Physical photograph (bottom and top side) of the designed neuromodulation system prototype.

Figure 6a gives the printed circuit board (PCB) layout illustration of the designed neuromodulation system prototype with four layers, where the SoC chip and auxiliary circuits are connected with two flexible layers (analog and digital wires). The power management circuits and designed SoC chip are placed on the bottom side while the data transmission circuits, a micro USB connector, and several passive components are designed on the top side. Figure 6b shows the physical photograph of the designed neuromodulation system prototype. In this design, the miniaturized prototype is developed for nerve recording and stimulation experiments in a small animal model.

### 4.2. Animal Surgery and Experimental Preparation

Animal experiments were performed in a sound attenuating, electrically shielded booth, and guinea pigs (450 ± 50 g; Elm Hill, Chelmsford, MA, USA) were used in accordance with the policies of University of Minnesota Institutional Animal Care and Use Committee. The animals were anesthetized with an intramuscular injection of ketamine (40 mg/kg) and xylazine (10 mg/kg) with 0.1 mL supplements every 45–60 min to maintain an areflexive state. Heart rate and blood oxygenation were continuously monitored via a pulse oximeter and body temperature was maintained at 38.0 ± 0.5 ${}^{\circ }$C using a heating blanket and rectal thermometer.

**Figure 7:**
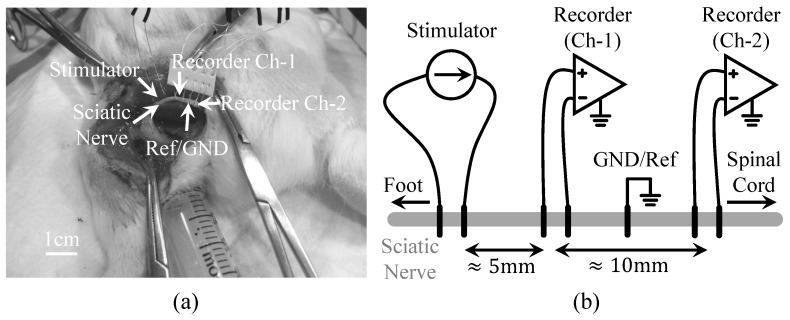
Nerve activity recording from a guinea pig’s sciatic nerve, where a two-channel fully differential nerve recording is performanced on the same nerve with current stimulation in a bipolar configuration. (**a**) Physical photograph and (**b**) illustration of experimental setup for simultaneous stimulation and recording.

In the nerve preparation as shown in Figure 7, the left sciatic nerve was exposed and separated from surrounding tissue. Two platinum wires (AS 770-36, Cooner Wire, Chatsworth, CA, USA) were wrapped around the exposed nerve distal to the spine for delivering constant current pulses (0.5 mA, biphasic, 500 μs pulse duration) to the nerve. Five silver wires (AS 766-36, Cooner Wire, Chatsworth, CA, USA) were used to wrap around the sciatic nerve proximal to the spine for recording. The middle silver wire was used as reference while the other four wires were used as two pairs of recording sites.

### 4.3. Animal Experimental Results from the Proposed Prototype

**Figure 8:**
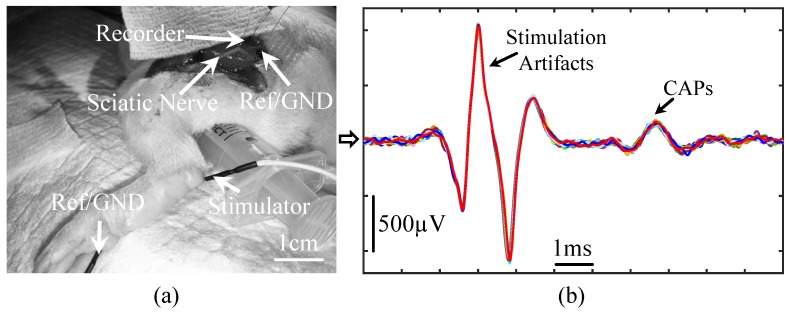
Compound action potentials (CAPs) recordings from a guinea pig’s sciatic nerve, where the stimulation current is presented to the guinea pig’s left foot and a one-channel fully differential nerve recording is performed on the sciatic nerve. (**a**) Illustration of experimental setup; (**b**) Recorded compound action potentials in response to foot stimulation with 2 mA, biphasic, 500 μs pulse duration current. In total, 30 trials are plotted, where each colored curve represents a single trial.

To demonstrate the proposed prototype is capable of acquiring CAPs, Figure 8 presents the recorded signals from a guinea pig’s sciatic nerve, where 2 mA, biphasic, 500 μs current pulses were presented to the guinea pig’s left foot to trigger nerve activity along the sciatic nerve. A single-channel fully differential nerve recording acquired the activity along the guinea pig’s sciatic nerve. The recorded nerve activity was filtered at 300–5000 Hz, and the peak-to-peak amplitude of the stimulation artifacts and CAPs is approximately 2.1 mVpp and 250 μVpp, respectively.

**Figure 9:**
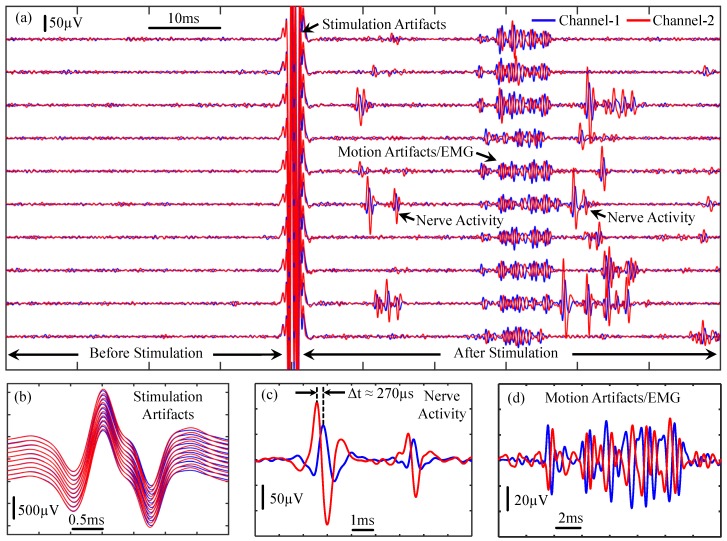
Measurement results of nerve recording and stimulating on the same sciatic nerve. (**a**) Ten trials of the recorded nerve activity showing the recorded waveforms in response to electrical stimulation of the sciatic nerve; (**b**–**d**) Zoom-in of the recorded stimulation artifacts, nerve activity, and motion artifacts/electromyography (EMG), respectively.

Figure 7a,b show the physical photograph and illustration, respectively, of the experimental setup for recording and stimulating on the same sciatic nerve. The nerve is exposed and in contact with platinum and silver wire electrodes. From left (foot) to right (spinal cord), we placed one stimulation channel and two recording channels, and the spacing between the two recording channels is about 10 mm. One way to verify whether the data consist of actual nerve activity (instead of muscle responses or other surrounding biological or artifact signals) is to confirm that the signals are propagating along the nerve, which is a challenging task. Figure 9a shows ten trials of the recorded waveforms in response to electrical stimulation on the same nerve, where 0.5 mA, biphasic, 500 μs current pulses were presented to the guinea pig’s sciatic nerve to trigger nerve activity. The signals include stimulation artifacts, nerve activity, motion artifacts, and electromyography (EMG) signals. The data were filtered at 300–5000 Hz. Figure 9b–d show the zoom-in of the recorded stimulation artifacts, nerve activity, motion artifacts/EMG, respectively. As shown in Figure 9, electrical stimulation can evoke neural responses. However, the stimulation artifacts can be quite large that are likely masking neural activity immediately evoked after stimulation onset. Note that there is no apparent delay in recorded stimulation artifacts across the recording channels, which is expected since the current flow spreads nearly instantaneously from the stimulation electrodes to both sets of recording electrodes. Interestingly, we observed nerve activity at about 8–20 ms after stimulation onset in which the recording channel closest to the spinal cord (channel-2, red) exhibited activity before channel-1 (blue; Figure 9a,c). This suggests that electrical stimulation may activate the sciatic nerve, which initially propagates towards the spinal cord. We may not have been able to detect those ascending signals because they were masked by the electrical artifacts. However, there are feedback signals from the spinal cord that propagate back from the spinal cord to the foot along the sciatic nerve, which would correspond to the spike activity shown in Figure 9a,c. There is about a 200–300 μs phase delay between the recorded spike activity from the two channel recordings, which is consistent with the conduction velocity expected for the sciatic nerve in rodents [38,39]. The EMG or muscle responses are likely elicited by the initial electrical stimulus activating the sciatic nerve down to the muscles rather than those feedback signals since the muscle activity exists even when there were no noticeable feedback signals. There does not appear to be any systematic delay between recording channels for the muscle activity, which is consistent with the two channels recording far-field muscle signals that reach both sets of electrodes nearly simultaneously. There also exists additional spike activity with longer delays after stimulation onset, which predominantly appears to originate from the spinal cord down the sciatic nerve since the activity appears more frequently on channel-2 before channel-1. Overall, these data demonstrate the powerful capabilities of our stimulation and recording system in sensing low amplitude neural signals and conduction delays in sub-populations of nerve fibers using simple non-invasive wire electrodes around the sciatic nerve, which is enabled by the low noise properties of our device. Further improvements are being pursued by our collaborative research group to better reduce and quickly suppress the electrical artifacts to detect any spike activity that may have been masked immediately after stimulation onset.

### 4.4. Animal Experimental Results from Commercial System

**Figure 10:**
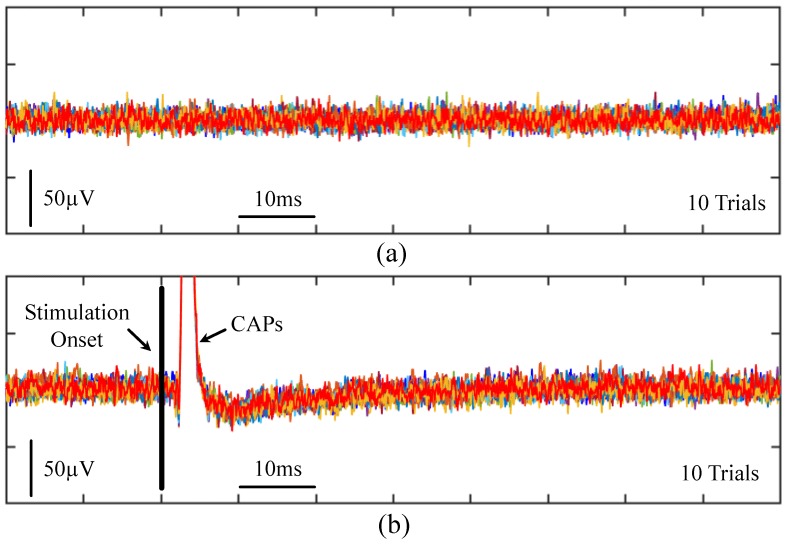
Measured nerve activity with a commercial system (Tucker–Davis Technologies (TDT), Alachua, FL, USA; PZ2-64 (Amplifier) and RZ2 (BioAmp Processor)) when the stimulation electrode is connected to the foot and the recording electrode is interfaced with the left sciatic nerve of a guinea pig. (**a**) Ten trials of the measured nerve activity when the stimulation electrode does not deliver current. (**b**) Ten trials of the measured nerve activity when the stimulation electrode delivers 2.82 mA, biphasic, 205 μs current pulses.

**Figure 11:**
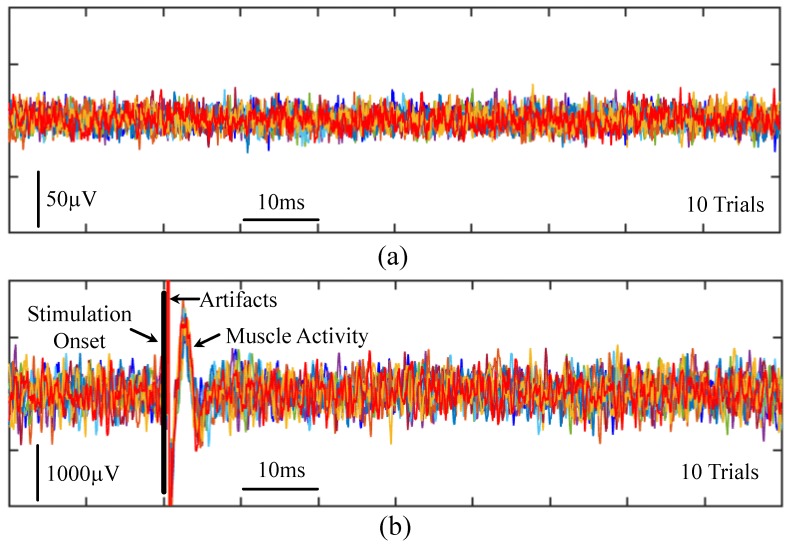
Measured nerve activity with the TDT system when both the stimulation electrode and recording electrode are connected to the left sciatic nerve of a guinea pig. (**a**) Ten trials of the measured nerve activity when the stimulation electrode does not deliver current; (**b**) Ten trials of the measured nerve activity when the stimulation electrode delivers 14.13 μA, biphasic, 205 μs current pulses. Note the drastic change in signal amplitudes and ordinate scale bars in (**b**) compared to (**a**).

To demonstrate the performance of the proposed neuromodulation prototype, we also compare the measurement results with one commercial system (Tucker–Davis Technologies (TDT), Alachua, FL, USA; PZ2-64 (Amplifier) and RZ2 (BioAmp Processor)). Figure 10 shows the measured nerve activity at 1.6–7500 Hz with the TDT system when using the same experimental setup as shown in Figure 7, where the stimulator is connected to the foot and the recorder is interfaced with the left sciatic nerve of a guinea pig, and the stimulation electrode delivers current pulses to the nerve. The measured noise floor without (Figure 10a) and with (Figure 10b) the electrical stimuli is similar, and its peak-to-peak value is ∼40 μVpp. Thus, the stimulation on the foot does not noticeably increase the noise floor of nerve recording. Figure 11 shows the measured nerve activity at 1.6–7500 Hz with the TDT system when using the same experimental setup as shown in Figure 8, where both the stimulator and recorder are connected to the left sciatic nerve of a guinea pig, and the stimulation electrode delivers current pulses to the nerve. Measurement results show the peak-to-peak value of the recorded noise floor without (Figure 11a) and with (Figure 11b) the electrical stimuli. The ordinate signal amplitudes and scale bars are drastically larger when the electrical stimuli are presented versus when no stimuli are presented (i.e., ∼1800 μVpp and ∼60 μVpp, respectively). It is clearly shown that presenting stimuli on the same nerve can increase the noise floor by 30-fold, even with high-end standard commercial physiology devices. Therefore, it has been very difficult for current neuromodulaiton technologies to simultaneously support electrical recording and stimulation on the same nerve, a challenge that we have begun to overcome with our neuromodulation system.

## 5. Conclusions

A bidirectional neuromodulation SoC has been proposed for simultaneous nerve recording and stimulation. Several methods have been proposed in our device design to reduce both electronic noise and electrode noise. First, we have pioneered a new switched-capacitor neural recorder architecture based on the frequency-shaping technique, which does not require any analog high-pass filter in the frontend circuits. As a result, the filter-related noise, artifacts, and distortions can be avoided through the frequency-shaping filterless neural amplification. Second, several noise optimization presented in Section 3.1 have been developed in the frontend circuits to reduce transistor thermal noise and switched-capacitor noise. Third, to reduce activity-dependent noise (shot noise) in the electrode interface, high input impedance is designed in the recorder to reduce current passing through the electrode interface. Fourth, because the recorder is implemented with switched-capacitor circuits, the electrode shot noise becomes zero when the recorder is disconnected from the electrode. Thus, the electrode noise can be minimized by adjusting the “electrode-on” duty cycle. These improvements in the design have enabled the proposed SoC to potentially detect nerve activity at a sub-10 μV range, even with electrical stimulation on the same nerve.

For verification, the designed SoC has been tested with two sets of animal experiments, in which the results demonstrate the SoC is capable of acquiring high-fidelity nerve activity along with stimulation artifacts, CAPs, and motion artifacts/EMG. Further improvements are being pursued to better suppress the electrical artifact during stimulation to enable more immediate recordings after stimulation onsent. The successful development of simultaneous recording and stimulation technologies for nerve interfacing applications with low-noise recording capability will open up new opportunities to study the function and interactions of nerves and organs, as well as advance clinical opportunities for precise treatment of various diseases and health conditions.

In the future, wireless and wearable transcutaneous power delivery and data telemetry will be required to enable the neural devices to be implanted and the subjects to move freely in their daily activities. To miniaturize the SoC as small as a grain of rice when incorporating all the circuits, the wireless power deliver and data telemetry would be also designed with custom VLSI circuits. In addition, new calibration algorithms and shielding methods are required to reduce wireless interferences. These technical challenges are currently being investigated by our research group.
